# Healthcare-induced trauma in correctional facilities: a qualitative exploration

**DOI:** 10.1186/s40352-021-00139-5

**Published:** 2021-06-21

**Authors:** Johanna E. Elumn, Layne Keating, Amy B. Smoyer, Emily A. Wang

**Affiliations:** 1 SEICHE Center for Health and Justice, General Internal Medicine, Yale School of Medicine, New Haven, USA; 2 Frank H. Netter MD School of Medicine, Quinnipiac University, Hamden, USA; 3 Department of Social Work, Southern Connecticut State University, New Haven, USA

**Keywords:** Healthcare, Trauma, Incarceration, Correctional facilities, Prison, Qualitative

## Abstract

**Background:**

While incarcerated people are known to experience trauma at higher rates than the general population, little is known about how the correctional health system contributes to trauma rates.

**Methods:**

We conducted 20 semi-structured qualitative interviews with men who were recently released from a correctional system to understand their experiences with healthcare systems and medical staff during incarceration. Using reflexive thematic analysis within a critical realist framework, we coded and analyzed the data iteratively to refine and unify emerging themes.

**Results:**

The unanticipated concept of healthcare-induced trauma emerged and was revealed in three overall themes: (1) healthcare leading to fear of serious illness or death, (2) healthcare leading to fear of people, including healthcare providers, correctional staff, and other incarcerated people, and (3) the correctional institutional, social, and physical environment leads to fear of place.

**Conclusions:**

Healthcare in correctional settings has the potential to induce trauma, even when the medical conditions addressed are not life-threatening. Future research should examine the factors contributing to the development of healthcare-induced trauma in correctional settings and develop interventions to prevent and address this phenomenon.

## Background

Almost by definition, incarcerated and formerly incarcerated people are survivors of interpersonal and structural trauma. Research has documented elevated rates of traumatic events and post-traumatic stress syndrome among justice-involved men and women (Moloney, van den Bergh, & Moller, 2009; Wolff, Huening, Shi, & Frueh, 2014). The events that lead up to incarceration often include interpersonal violence, extreme stress and anxiety, and institutional and structural violence (i.e., educational failure, homelessness, poverty) (Jäggi, Mezuk, Watkins, & Jackson, 2016; Sugie & Turney, 2017). Further, the incarceration experience itself – the social isolation, harsh conditions, exposure to physical violence – can be painful and traumatic (DeVeaux, 2013).

Healthcare, including medical care and mental health services, offers a remedy to this trauma. Doctors, nurses, and mental health providers can facilitate healing and recovery by mending broken bones, stitching tears, dressing wounds, and addressing the impact of emotional trauma. In fact, incarcerated people are among the only populations in the United States with a constitutionally guaranteed right to health care. Since 1976, *Estelle v. Gamble* has required that correctional facilities provide basic healthcare for acute conditions. In this seminal case, the Supreme Court ruled that refusal of these services constituted “cruel and unusual punishment” under the Eighth Amendment. This case then set precedent for other court cases which further expanded the right to healthcare: *Bowring v. Goodwin*, established that denial of psychiatric treatment was also considered “cruel and unusual.” As a result, people in prison are entitled to mental health treatment if they have symptoms a medical professional has deemed serious and curable, and there is substantial potential for harm if care is denied or delayed (Klein, 1978). These constitutional protections have meant that many people first access physical and mental health care while incarcerated, engaging in care for health conditions that were undiagnosed, unmonitored, or untreated in the community (Meyer et al., 2014).

In spite of these legal protections, access to quality healthcare and treatment of trauma is still a challenge for incarcerated people. The large number of people who are incarcerated in the United States and the aging of the nation’s prison populations make correctional healthcare very expensive (Ahalt, Trestman, Rich, Greifinger, & Williams, 2013; Fiscella, Beletsky, & Wakeman, 2017; Rich, Allen, & Williams, 2015; Williams, Stern, Mellow, Safer, & Greifinger, 2012). Underfunded medical services result in delayed services, poor quality healthcare, and negative interactions with correctional and medical staff, prompting some to develop strategies to cope, including educating themselves about their health conditions (Novisky, 2018; Visher, Naser, Baer, & Jannetta, 2005). Untreated and undertreated conditions can result in distressing experiences, including opioid withdrawal, debilitating infections, medication delays, and chronic pain issues (White Hughto et al., 2018; Smoyer, Elumn Madera, & Blankenship, 2019). Existing research has documented that interactions with medical staff are a primary stressor for incarcerated individuals, and in fact can be more emotionally upsetting than negative interactions with other correctional staff or peers, possibly due to expectations of the care they should receive (Porter, 2019).

While many studies describe problems with healthcare during incarceration, the current study expands on these earlier studies by examining how correctional healthcare systems might actually be a source of new trauma among incarcerated people rather than healing. For someone who is incarcerated, unaddressed health concerns, chronic pain, and the anxiety and fear of seeing others get sick and die may be traumatic and have long-lasting symptoms and health outcomes as documented in other health care settings. For instance, Krumholz (2013) has documented a phenomenon in hospitals, which he calls “post-hospital syndrome,” where medical issues coupled with the stress of receiving or not receiving care, creates a lingering effect after the initial health problem is resolved. Given the conditions of healthcare faced by incarcerated people, correctional facilities may produce a similar psychological impact. These issues are particularly relevant and compelling in light of the COVID-19 pandemic which has disproportionately impacted incarcerated populations. Existing mistrust of the healthcare system during incarceration and fears around lack of treatment, segregation, punitive responses, may complicate incarcerated people’s responses to COVID-19 treatment and vaccine acceptance.

### Understanding healthcare-induced trauma

This exploration about the impact of healthcare-induced trauma on incarcerated people is grounded in a broader conceptualization of trauma. Substance Abuse and Mental Health Services Administration (2014) defines trauma as:

Individual trauma results from an event, series of events, or set of circumstances that is experienced by an individual as physically or emotionally harmful or life threatening and that has lasting adverse effects on the individual’s functioning and mental, physical, social, emotional, or spiritual well-being …. Events and circumstances may include the actual or extreme threat of physical or psychological harm or the withholding of material or relational resources essential to healthy development. These events and circumstances may occur as a single occurrence or repeatedly over time. (p. 7–8)

The SAMHSA definition underscores that the person’s perception of the event, and not its actual severity, determines whether or not this event is experienced as traumatic and has a lasting effect on the individual’s functioning. People can experience something as traumatic that they directly experience, witness, or even learn about (American Psychiatric Association, 2013). Further, exposure to a traumatic event can result in symptoms that impair functioning, even if the person does not meet criteria for post-traumatic stress disorder (PTSD) (Herman, 2015; Van der Kolk, 2015).

The literature on healthcare-induced trauma provides a framework for understanding the effect of correctional health care on incarcerated people. Healthcare-induced trauma, also referred to as medical trauma, is defined as trauma that occurs as a result of a medical event that either subjectively or objectively results in pain, injury, or fear of death; can have ongoing implications for daily functioning and adherence to healthcare; and can impact recovery from the health crisis. Hall and Hall (2013) identified three overarching characteristics of the medical setting which contribute to the development of trauma: healthcare, the environment, and the patient’s mental health and the resources available to them. They posit that it is the combination of these three factors that determine how the experience impacts the patient and suggest interventions in each area that could mitigate the effects of the experience. Previous research on healthcare-induced trauma has focused on people’s reactions to specific medical events or conditions like myocardial infarction, HIV diagnosis, obstetrical complications, and intensive care scenarios (Hall & Hall, 2013; Jackson et al., 2007; Lerwick, 2016; Tedstone & Tarrier, 2003). While healthcare-induced trauma generally happens during hospitalizations, we propose that it can also occur in outpatient clinic settings when patients experience extreme stress related to health problems and healthcare.

**Fig. 1:**
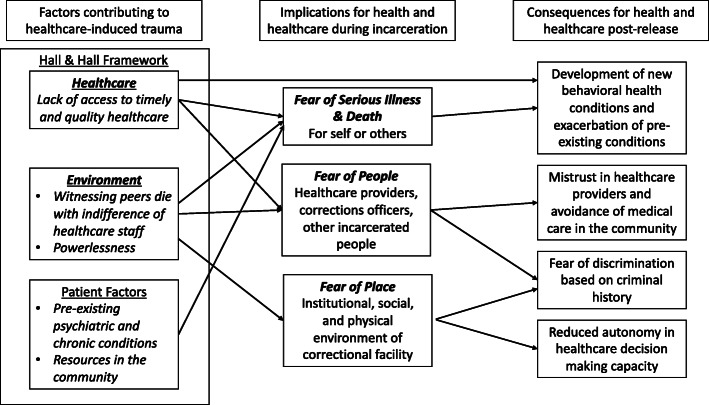
Hall and Hall (2013) Conceptual Framework Adapted for Use in Populations Released from Correctional Facilities

In this analysis, we build on the Hall and Hall (2013) framework (see Fig. 1) and highlight the ways in which this trauma is produced not just by events or diagnosis, but by the particular institutional context of correctional healthcare systems. Specifically, we explore how the prison environment and regulations amplify trauma in a population that is particularly vulnerable because of pre-existing mental health issues and often lacking the supports and access to care. We focus on the contributing factors of the carceral environment, including healthcare systems, rather than individual patient characteristics, because these institutional characteristics are more readily modifiable by changes to correctional structures and policies. These findings can inform correctional health policy and programs and also support work with formerly incarcerated people by explicating the ways in which healthcare-induced trauma in correctional facilities may influence engagement with medical professionals during and after incarceration.

## Methods

This study was part of a larger project that examined relationships between men’s experiences of healthcare during incarceration, trauma history, and utilization of primary care after release. Trauma research in incarcerated populations has historically focused on trauma that occurs in the community, childhood abuse, and experiences of women, adolescents, and aging prisoners (Cimino, Mendoza, Thieleman, Shively, & Kunz, 2015; King, 2017; Maschi, Gibson, Zgoba, & Morgen, 2011). We focused on the experiences of men because there is limited research on the trauma experiences of men during incarceration. As a part of this mixed-methods study, we conducted semi-structured qualitative interviews with men who were recently released from a correctional facility to understand their experiences with healthcare during incarceration. We used qualitative methods in order to center the voices of those who have been incarcerated and create space for the multifaceted complexity of trauma and healthcare seeking behaviors to surface (Braun & Clarke, 2014; Sacks, 2015). While the overall study did include a focus on traumatic experiences during incarceration, the concept of healthcare-induced trauma had not been articulated a priori and emerged from the interviews. Our analysis here focuses on data related to this unanticipated and significant issue which we feel demands specific attention separate from the other overarching themes in the interviews.

Our interview team was composed of the principal investigator, two formerly incarcerated men, one who had served 15 years in a state prison and another who had served 35 years in the federal prison system, and 2 s-year medical students. All team members were trained in human subjects research, basic research methods, and qualitative research, including interview guide development and qualitative interviewing techniques. All five participated in the development of the interview guide, administering the interviews, and one medical student and one of the incarcerated men participated in data dissemination through conference presentations. Prior to the start of the study, the team completed mock interviews that were recorded and transcribed and received feedback on those interviews to prepare them to conduct the study interviews. Due to the re-incarceration of one team member, scheduling conflicts, and time restraints of publishing results, only the principal investigator and one medical student participated in data analysis.

### Sampling

Using a purposive sampling strategy, we recruited 20 men recently released from a correctional system who were receiving services at a community-based primary care program in New Haven, CT that provides targeted primary care for people with histories of incarceration and serves approximately 100 new patients each year. Between September 2018 to March 2019, we interviewed participants who met the inclusion criteria; 18 years of age or older, male, spoke English and reported a history of trauma.

Specifically, a letter of invitation that described the study was shared with any participants who reported a history of trauma (not specific to incarceration or healthcare, but any form of trauma in general) to a member of the clinic staff. Participants were not asked about their experiences of healthcare during or after incarceration during the screening process. If they were interested in volunteering, study staff would contact participants to screen for eligibility. All participants who met criteria provided written informed consent and received $30 for completing the interview. In order to create a sample that reflected the race and ethnic diversity of the clinic’s client population, recruitment was stratified by race. Of the 21 men asked to participate, only one was unable to participate because of serious illness at the time of recruitment.

### Data collection

Those who agreed to be interviewed completed a brief survey prior to the interview about their incarceration history and mental health diagnoses. The semi-structured interview instrument contained nine open-ended questions focused on healthcare during incarceration, trauma during incarceration and in the community, coping with trauma, and healthcare after release. All participants were individually interviewed in private offices where the research team was based. The interview guide included non-standardized prompts to provide clarification of the emerging concepts in the interviews (Patton, 1990). All interviews, which lasted approximately 90 min, were digitally recorded and transcribed using Rev.com. The transcripts were reviewed by project staff and then imported into qualitative data management software (Dedoose) for analysis.

### Data analysis

All interviews were coded by the first author and second author and analyzed using reflexive thematic analysis (Braun & Clarke, 2006, 2014). Our approach to thematic analysis was an inductive and descriptive approach that engaged in a critical realist framework, which considers the context and subjectiveness of people’s experiences and uses empathic interpretation, asking the researcher to put themselves in the position of the participant and interpret the interviews from their perspective (Fletcher, 2017; Madill, Jordan, & Shirley, 2000; Terry, Hayfield, Clarke, & Braun, 2017; Willig & Rogers, 2017). This approach to qualitative analysis highlights the voices of the men who participated and acknowledges the social and cultural context in which they experienced healthcare, both in prison and in the community (Braun & Clarke, 2006; Clarke & Braun, 2017). We used an inductive process that involved reading through the transcripts to become familiar with the content. We generated the initial codes and then reviewed and revised them. We drew on the healthcare-induced trauma literature and, in particular, the conceptual framework adapted from Hall and Hall (2013). The codes were then reviewed and patterns in the codes were identified as themes and those themes were reviewed and defined (Braun & Clarke, 2006). The names of the study participants were changed in this manuscript to protect their identity since the topics discussed were sensitive. The study was approved by the Institutional Review Board at the first author’s university.

## Results

Study participants were a diverse group of adult men who had extensive interaction with criminal legal system. The mean age of participants was 46 years and they ranged in age from 23 to 58. Forty percent of participants identified as Black, 40% as Hispanic/Latino, and 20% as White. All participants had significant experiences with incarceration; 63% experienced incarceration as a juvenile, the average number of sentences served was 6.4, and the average length of the most recent sentence was 11 years. Almost all participants (95%) had spent some time in solitary confinement, ranging from a total of 14 days to 15 years. Almost half of the participants (45%) had been diagnosed with PTSD and two or more psychiatric conditions. The men described their experiences being incarcerated in local jails, state prisons, and in federal correctional facilities.

### Healthcare-induced trauma

Participants described their own experiences with healthcare during incarceration, witnessing the experiences of others, and learning about what has happened to others either in the present or stories of past experiences. In this setting the exposure to trauma is extreme for several reasons: 1) repeated exposure to various forms of healthcare-induced trauma, 2) collective exposure, and 3) being in captivity and the loss of control that this experience entails. The severity and frequency of this trauma on an individual level might be modulated by underlying health conditions and length of time or frequency of incarceration as described in Fig. 1, but even participants without health concerns described experiencing healthcare-induced trauma. One participant when describing his struggle to get healthcare said, “because you don’t sign up for that”, indicating that the punishment of incarceration should not include the risk of losing his life. In this setting, incarcerated people bear witness to the deteriorating health and healthcare experience of others in a way they might not in the community. Even when someone does not directly experience or witness the health issues of another, they may hear about it. New stories of those with negative outcomes may be more likely to circulate in the facility and are compounded by stories of past incidents. Of note, these experiences were specific to healthcare within the correctional facility. A minority of participants had experiences with community healthcare during incarceration (namely being transferred to an outside hospital during their incarceration), but this was usually viewed positively as it resulted from a medical issue becoming more serious after being ignored or not treated appropriately by correctional healthcare staff. Experiences with community healthcare post-release were limited in this sample as participants were recently released. Although we did not ask specifically about the length of time since release, the men noted that they had limited contact with healthcare providers after release.

Central to the concept of healthcare-induced trauma is fear, and this was the feeling that was expressed most often in these interviews. Without the presence of fear, trauma does not materialize. Here we provide an example passage from a single participant who encapsulates healthcare-induced trauma and the fear that manifests as a result of both vicarious (witnessing and learning about) and direct experiences of healthcare.

Outlined earlier in detail, the Hall and Hall (2013) model identified three elements of healthlcare settings which contribute to the development of trauma: healthcare, the environment where the care takes place, and the patient’s preexisting mental health and resources. Several themes related to healthcare-induced trauma emerged in our study, namely (1) healthcare leads to fear of serious illness or death, (2) healthcare leads to fear of people (healthcare providers in jail/prison, correctional staff, other incarcerated people, community medical providers), and (3) the correctional institutional, social, and physical environment leads to fear of place (Table 1). These fears fueled and were fueled by feelings of desperation, anger, mistrust, and powerlessness connected to lack of access to timely and quality healthcare. Fear of place included participants’ descriptions of aspects of the prison environment that contributed to trauma.

**Table 1:** Healthcare-induced Trauma by Theme

Theme	Illustrative Quotation
Theme #1: Healthcare leading to fear of serious illness or death	“Kind of depressing when you think that they’re [medical staff] to help you and they don’t. I even called my family, because I was nervous that I was going to die.” (Tyler, Age 44)
Theme #2: Healthcare leading to fear of people (including healthcare providers, correctional staff, and other incarcerated people)	“It was the general idea that if you go up there [to Medical], they’ll kill you. Like they just don’t care.” (Dwayne, Age 31)
Theme #3: The institutional, social, and physical environment of correctional facilities leads to fear of place	“It’s just bleeding the vile and the years of- of disease and infestation and people coughing and not covering it, not having the respect for each other, and you know, picking their nose and out it everywhere and just- it’s terrible.” (Marcus, Age 55)

These experiences of trauma related to their health, their interactions with healthcare providers, and the prison environment intersected with each other to create a compound experience of trauma that was greater than the sum of its parts (Crenshaw, 1993). In the words of one participant, Jose, the disordered correctional healthcare system created a “snowball effect” that he experienced as traumatic:

Your health is going down and what kills you is the psychological part because you sit in there in the box and you like you know am I gonna die? … then this dude next door in the cell he died of colon cancer. They told him if he would have checked this six months [ago] that he would have lived. You know that's how prison work … So everything stopped working, I started losing weight, I started getting my people nervous out here, I start bugging out in there … So it brings problems with your cellmate. Cause now you bringing all that frustration, he got frustration. Everything starts. It's like a snowball effect. (Jose, Age 41)

This narrative articulates a clear connection between how healthcare-induced trauma, namely lack of access to quality care and witnessing peers die, creates complex fear and mistrust and results in worsening mental and physical symptoms. All three elements of fear that surfaced in this data are present. He expresses fear of death, “am I gonna die?;” he articulates a profound distrust and fear of the medical providers by asserting intentionality, “that’s how prison works;” i.e., the health care providers are not actually there to heal; and the place breeds terror: It is not only that Joseph is feeling sick, it is that he is being held in solitary confinement [“in the box”] with people “next door” who are dying from lack of treatment. Further, the impact is far-reaching, breeching the walls of the prison to disturb his “people” in the community and causing interpersonal conflict within the walls: “Everything starts. It’s [health problem] like a snowball effect.” The sections that follow lay out the factors that contribute to healthcare-induced trauma and all portray different aspects of this type of trauma.

### Healthcare leading to fear of serious illness or death

Fear of serious illness or death emerged out of participants’ experiences of healthcare and witnessing or learning about the healthcare experience of other men incarcerated with them. Participants reported that it was common for medical staff to misdiagnose and minimize health problems. This lack of recognition or validation of health problems created considerable fear that problems would go untreated and lead to poor outcomes, including death. In this passage, Sean describes being repeatedly dismissed by medical providers and only gaining access to care when he was “really in bad shape.”

I went to sick call probably four or five times and was told just to drink water and buy cough syrup from the commissary. Eventually, I went over the head [of the provider] but not until I had lost 30 pounds and was really in bad shape … really trying to hang on by the skin of your teeth until you could finish the [prison] sentence. (Sean, Age 51)

This image of “hanging on” illustrates the desperation of incarcerated people trying to seek care and hoping they can survive long enough to be released. Sean described how he was immediately sent out to the hospital with pneumonia after turning to the hospital administrator for help. Similarly, Tyler described an absence of care that left him feeling depressed. He reported relying on those incarcerated with him, instead of medical providers, for medical assistance. He became so “nervous” about his possible death that he called his family to alert them “if something ever happens.” His narrative speaks directly to a fear of death provoked by lack of medical attention:

Yeah, I had to have another inmate carry me to the bathroom, help me, walk me, get me some drinks when I needed it, stuff like that. Kind of depressing when you think that they're [medical staff] to help you and they don't. I even called my family, because I was nervous that I was going to die. So I let them know... “If something ever happens, this is what's going on.” (Tyler, Age 44)

This trauma was also brought on by seeing friends’ experiences with healthcare. Marcus, for example, watched his friend struggle with the prison’s heathcare system which continually dismissed his concerns: “medical wouldn’t help him … This process lasted for a couple of years, and it’d usually bring tears to my eyes to see him suffering. And then people didn’t want to, you know, don’t want to sit down and eat with him.” The emotional toll of watching other people suffer was traumatic: “Affect me? It’s the trauma. I cared about [him] for a long time.” (Marcus, Age 55) Much of this fear emerged from feelings powerlessness around managing their health conditions and accessing the care they needed, further augmenting the trauma.

### Healthcare leading to fear of people

Participants also reported that their experience of healthcare also led to fear of people including healthcare providers, correctional staff, and other incarcerated people. Jose described the stress of being at the mercy of staff to access healthcare and compared it with the violence he had been exposed to during childhood and while incarcerated.

...The health thing really affected [me] more. Cause you really can't do nothing about it, but the violence you can really can resolve, you really can act out violence towards anybody and really kind of get that out your heart. Health you really stuck, you really can't, you can't force nobody to take you to the doctor. You might could go to see a nurse through the door or something, but it's different. You’re powerless. (Jose, Age 41)

His words illustrate the impact that this powerlessness around his health had on him psychologically. Even in violent situations in the community and during incarceration there were ways that you react or cope and “get that out your heart.” In the community, you could change providers or go to the emergency room, but inside you “can’t force nobody to take you to the doctor.”

Many of the men pointed out that in the prison environment the line between corrections officers and medical staff can blur, and medical staff can have real or perceived dual loyalty to patients and prison authorities. Miguel described the prison administration and medical staff as untrustworthy. The neglect he witnessed made him fear for his own health:

In the federal system, I don't trust the administration and I don't trust the medical because I done seen too many people died when they wasn't supposed to die. .... They went there with stomach problems, they kept telling them it's just a virus, it's just a virus. You know two people end up dying cause they had stomach cancer and they wasn't addressing it. So yeah, I was scared. (Miguel, Age 47)

From Dwayne’s perspective, even a legitimate medical concern was not enough to convince most of his peers to attempt to utilize medical services.

Um, a whole bunch of people was like, “Shit, I'd rather just sit here and die at the sewing machine than go to medical because then what?” … it was the general idea that if you go up there, they'll kill you. Like they just don't care. (Dwayne, Age 31)

Fear of retaliation by others incarcerated with them and of punishment by correctional staff kept people from seeking care when they need it, even if they are in severe pain, as highlighted by Rob:

And, you know, the environment is, I can't let you know that that affected me. I can't cry in front of you. I can't show weakness in front of you because now I become the prey. So everybody got to laugh it up. I don't need to talk to no damn body, what? I seen shit like that all the time. When in reality it's tearing you up inside … But who could, you can't go to medical, because medical's going to tell on you. (Rob, Age 57)

Concern that the medical staff would not treat you or would cause further harm were at the forefront of the minds of these men and their peers when a health issue surfaced and led to hypervigilance around avoiding going to the medical unit for treatment.

Regarding community healthcare, our participants generally described positive experiences with the community-based primary care office targeted towards patients with a history of incarceration, but most had not interacted with other healthcare services due to their recent release. A few participants described avoiding healthcare due to fear of discrimination because of their history of incarceration.

### The institutional, social, and physical environment of correctional facilities leads to fear of place

The prison environment, both the physical environment and the social structures of prison life, contributed to the way the men felt. The fear of experiencing administrative or housing problems if they complained about their health was a recurrent concern. Their health issues were ignored at baseline, but they risked being moved to an environment where if something went wrong it might go unnoticed until it was too late. Luis expressed having to decide between accessing healthcare for asthma or avoiding punitive action.

… the whole time I was there it was just one big argument just to get an asthma appointment. It was either … talk shit to them and basically get to the point that they're gonna write me up for threatening them, and like we gonna lock you up in the cell because I'm, I'm, I'm trying to address my, my medical issues and all they was doing was stressing me out. The response was, “Don't talk like this, don't even do this, matter of fact we'll send you back to your cell, give you a ticket... and don't come down here no more. (Luis, Age 55)

The men voiced that tickets or punishments only put them at even greater risk of serious illness or death, as segregation limited their contact with peers and staff and loss of good time meant you had to spend more time in an environment where you were at risk. At the same time, actions like avoiding the medical unit was also a way that these men took back control over what happened to them. At times they choose to suffer with pain, turn to other incarcerated men for help, and reach out to family in the community to get the help they needed, all forms of resistance that the men employed.

Fear and desperation around their health caused by a lack of institutional response from correctional healthcare is traumatic:

You really do get scared because, you never know if one day you might go through something that's life threatening and they have no solution for you. So it's like now your down at medical and you're feeling like you're damn near on your deathbed and it's like, the only thing they can tell you is, “Oh you'll be all right, here's some Motrin.” Or, “You'll be all right, just sleep it off and come see us tomorrow.” So now it's like, what if you do go back and that's your last sleep? (Dwayne, Age 31)

For Dwayne and many those we interviewed anxiety around their health and the lack of healthcare was compounded by the prison environment and led to hypervigilance around any health issue that came up. Since they could not trust that the medical staff was acting in their best interest, they became fearful of anything that might indicate a health problem, even something minor. A cough might just be a cough, or it might be lung cancer and lack of treatment might ultimately lead to death. In our interview with Marcus, he described the unsanitary conditions of his facility at length and in vivid detail. There was a clear connection in his mind between these surroundings and getting sick, and he became fixated on his hygiene to the point that he started making and selling his own soap and doing laundry for his peers.

You keep painting over fungus and it's just peeling through the walls. If you touch the walls you can feel it biting you. It gets on yeah- it's just- it's just bleeding the vile and the years of- of disease and infestation and people coughing and not covering it, not having the respect for each other, and you know, picking their nose and out it everywhere and just- it's terrible. (Marcus, Age 55)

Marcus’ comments highlight the way the physical environment of the prison, the lack of cleanliness and the inability of the men to control this, contributed to their fears. His description is haunting with the “bleeding” walls and the “years of disease” that cover them in layers. Not only might they be ill, but the environment might be contributing to their sickness or preventing them from being able to heal both physically and mentally. In this description of the physical environment, the walls are a constant reminder of illness and trauma.

Sean highlights how many of the men perceived the prison environment as intentionally discouraging them from accessing healthcare by making it difficult to access care, providing minimal care (e.g., only offering Motrin), or sending them away with no resolution..

Other places would be so nasty and so mean and so um, you know, bare minimum that you wouldn't even wanna go to medical. And they liked it that way. They created it that way. They would create an atmosphere to where they would ... Guys would say, “Uh, I'm not even gonna bother cause they're not gonna do nothing for me anyway.” (Sean, Age 51)

By providing minimal or no healthcare, some prisons “created an atmosphere” where the men did not bother to go to the medical unit because they knew they would not receive treatment.

## Discussion & Implications

While existing research has demonstrated that incarcerated people often having complex histories of trauma and may be exposed to physical and psychological trauma during incarceration, the phenomenon of healthcare-induced trauma during incarceration has been underexplored. Using the framework created by Hall and Hall (2013), we reveal how experiences with healthcare systems and personnel, combined with the harsh prison environment, produces a healthcare-induced trauma that has not previously been described among people involved in criminal legal system. The institutional, social, and physical environment of correctional facilities contribute to these traumatic experiences. Participants reported that the lack of medical attention caused them to fear for their lives while incarcerated. Even when a person’s health issues were not life threatening, the dismissal of their concerns by providers caused significant pain and distress (Davey, Clarke, & Jenkinson, 2019). They were fearful and distrustful of healthcare professionals, correctional officers, and administrators. The prison place was experienced as filthy and infected. This healthcare-induced trauma was further compounded by the vicarious trauma from witnessing peers become ill and die, and carceral experiences of violence, noise, light, staff discretion, and unsanitary conditions. Healthcare-induced trauma highlights the structural violence of incarceration, which results in “the imposition of unequal risk for disease, injury, and death by social, political, institutional, and economic configurations and policies on identifiable population groups” (Karandinos & Bourgois, 2019, p. 6).

This data suggest that healthcare-induced trauma experienced in correctional facilities can have a lasting effect on an individual’s relationship with healthcare systems. Participants described how traumatic healthcare experiences provoked a lack of trust in correctional healthcare staff. This distrust may corrode the patient-provider relationship and trigger avoidance of healthcare services in both correctional and community settings, leading to worsened health outcomes for the individual. Previous research has shown that those who have experienced incarceration were less likely to see a primary care provider for a health problem and are more likely to use acute care services, are less likely to receive oral healthcare services, and are more likely to face barriers to prenatal care when compared with those never incarcerated (Kulkarni, Baldwin, Lightstone, Gelberg, & Diamant, 2010; Testa & Fahmy, 2020; Testa & Jackson, 2020). While the origin of these patterns of healthcare use is likely multifactorial, including discrepancies in financial resources, health insurance status, fear of police surveillance or even arrest, and perceived discrimination because of incarceration status, a cycle of fear and avoidance of healthcare resulting from healthcare-induced trauma may be another contributory factor (Frank, Wang, Nunez-Smith, Lee, & Comfort, 2014; Brayne, 2014).

Our findings have implications for correctional healthcare policies and programs. Improving correctional healthcare will require many system- and institution-level changes, including implementing and enforcing universal standards, increasing funding, and improving training. By addressing healthcare-induced trauma in all of these domains, in contrast to other sources of trauma, there is the opportunity make systemic changes in order to prevent this traumatic stress from occurring (Weinert & Meller, 2007).

Efforts to shift institutional climate and communication are relatively inexpensive, yet organizationally complicated to implement. For example, prohibiting the use of solitary confinement and other forms of punishment in response to requests for healthcare would help to prevent associations between healthcare and trauma. Communicating with incarcerated people that their requests for medical assistance have been received and offering at least a broad timeframe for response, could reduce the sense of desperation articulated by participants. Providing professional development and training for both correctional officers and healthcare staff about reflective listening could improve staff capacity to respond in meaningful ways to individual’s concerns; the relatively simple act of validation (I see you, I hear you- can offer relief) (Kubiak, Covington, & Hillier, 2017; Levenson & Willis, 2019; Miller & Najavits, 2012). Similarly, building restorative circles that allow incarcerated people and medical providers to communicate about the challenges they have both faced could improve communication and build trust (Thomas, Bilger, Wilson, & Draine, 2019). Finally, correctional facilities might develop a community health workers model, as has been done outside prison, to train incarcerated people to help peers navigate the healthcare system and build trust (Morse et al., 2017; Shavit et al., 2017). Given the reality of shrinking state and federal budgets, peer education programs offer facilities the opportunity to harness the power and skills of incarcerated people to improve the prison healthcare environment.

With adequate funding, the menu of possibilities expands considerably. Improved access to mental health services in correctional settings can 1) provide a space for individuals to share and process experiences of healthcare-induced trauma, 2) examine and address prior trauma and psychiatric comorbidities, and 3) develop positive coping skills and resilience. As described earlier, addressing trauma in correctional settings can be difficult, but there is research discussing the utilization of trauma-informed care practices in these institutions, primarily with female and juvenile populations (Branson, Baetz, Horwitz, & Hoagwood, 2017; Harner & Burgess, 2011; Miller & Najavits, 2012). In order for these services to be utilized and effective, correctional facilities must also address issues of stigma and confidentiality individuals might face by accessing mental health services.

Support for medical staff is also key. Mental healthcare and training is needed for medical staff who are working to survive the same violent, under-resourced environment that participants describe. Issues of dual loyalty, or the extent to which healthcare staff work for the prison or the patient, require careful attention and discussion in order to maintain staff commitment to their patients and avoid the burnout that can lead to issues of poor care and lack of trust between providers and patients in correctional settings (Glowa-Kollisch et al., 2015; Pont, Stöver, & Wolff, 2012). Central to improving healthcare in correctional facilities is addressing the assumptions held by correctional staff that patients are voicing false health concerns or malingering. Bringing the results of this study to correctional administrators, medical staff, and corrections officers is one step in starting a conversation around these assumptions and healthcare during incarceration. Supervision of medical staff led by medical professionals outside of the correctional hierarchy would aide in this effort to eliminate dual loyalty (Pont et al., 2012).

Finally, trauma-informed care (TIC) could be implemented in both correctional settings to improve patient outcomes and also in the community practices situated in communities with high rates of incarceration. TIC practices have evolved since their introduction over 30 years ago and may be implemented differently in certain settings or with specific patient populations. Typically, TIC involves 1) maximizing patient safety, both real and perceived, 2) empowering patient autonomy and resilience, and 3) improving patient-provider working alliance through identifying patient-specific impact of trauma history in a compassionate and culturally competent manner (Wilson, Pence, & Conradi, 2013). TIC in the medical setting has largely been implemented and researched in patients with sources of primary trauma such as sexual assault and intimate partner violence (Reeves, 2015). However, there is a recent push to include more TIC practices in medical settings like the intensive care unit (ICU), neonatal intensive care unit (NICU), and pediatric emergency medical services (EMS), to target healthcare-induced trauma specifically (Ashana, Lewis, & Hart, 2020). Researchers have recommended implementing TIC strategies into the ICU setting to address both pre-existing trauma and the traumatic nature of critical illness and the ICU setting itself (Ashana et al., 2020). The patient’s heightened arousal, and even hypervigilance, can impact decisions about care, lead to patients or their family members being labeled as “difficult”, and impair healing. These strategies can also be applied to correctional settings, but there are significant structural challenges to using this strategy in places where security may be in conflict with providing TIC (Miller & Najavits, 2012).

This study had several limitations. First, the nature of qualitative research suggests that reported experiences might be subject to recall bias. Secondly, our participants were 20 cisgender men living in Connecticut with a history of trauma unrelated to healthcare who were patients of a clinic designed specifically to provide primary care to people after release. Therefore, their experiences may not be shared by those with other characteristics or incarcerated in different facilities or locations and may differ from other recently released people who never sought out healthcare after release. Being able to incorporate the perspective of someone with a history of incarceration into the coding and analysis process could have informed our interpretation of the response to correctional healthcare.

## Conclusions

This study illuminates how healthcare experiences during incarceration can be a source of enduring trauma which impacts psychosocial and health outcomes. The participants’ struggles to receive timely and quality healthcare, the loss of other men they were incarcerated with, and the trauma they faced as result of these experiences demonstrate that the mandate of *Estelle v. Gamble* has not been achieved. Future research should continue to investigate how correctional healthcare is a source of trauma and its specific consequences for health and healthcare post-release. Especially important and urgent, given the millions of people exposed to healthcare in corrections daily (and especially during the COVID-19 pandemic), are studying effective strategies for mitigation or prevention.

## Data Availability

The data from this current study available from the corresponding author on reasonable request. The sensitive nature of the topics discussed in the interviews are a consideration in releasing any data. The details participants provided in the interviews could lead to the identification of the study participants and this will be considered in any requests for the data.
